# Retrograde tracheal intubation using the S‐guide intubation guide after emergent tracheotomy: A case report

**DOI:** 10.1002/ccr3.9294

**Published:** 2024-08-14

**Authors:** Fiona Ollier, Alain‐Stéphane Eichenberger, Georges L. Savoldelli, Julien Maillard

**Affiliations:** ^1^ Division of Anesthesiology, Department of Anesthesiology, Pharmacology, Intensive Care and Emergency Medicine Geneva University Hospitals Geneva Switzerland

**Keywords:** airway management, anesthesia, difficult intubation, retrograde intubation

## Abstract

Retrograde intubation is an historic technique that is now rarely used in difficult airway situations. Although not originally designed for this purpose, the use of an S‐guide is a feasible option to aid retrograde intubation because of its malleable rigid body and atraumatic tip.

## 1 INTRODUCTION

“Cannot intubate, cannot oxygenate” (CICO) is a rare complication of airway management but remains a major cause of death associated with general anesthesia.^1^ All national airway guidelines recommend front‐of‐neck access (cricothyroidotomy) as a rescue technique in this life‐threatening condition.^2^, ^3^, ^4^

After achieving rescue oxygenation and ventilation via cricothyroidotomy, physicians may consider different scenarios depending on the etiology and required duration of a secured airway. Tracheotomy may be indicated, or early neck closure might be desirable. In such cases, restoring the proper airway with oro or nasotracheal intubation through fiberoptic intubation is the least invasive method. However, gross anatomical upper airway deformities or active bleeding can make video‐assisted or fiberoptic intubation nearly impossible, even when performed by skilled personnel. In these situations, an alternative method, known as retrograde intubation, may be considered. In this case, we describe retrograde tracheal intubation using an S‐guide intubating guide [VBM Medical] as an alternative to the central venous catheter guidewire after urgent tracheotomy for CICO.

This case report adheres to the Case Report (CARE) guidelines for clinical case reports.^5^ The patient provided written informed consent for this study.

## 2 CASE HISTORY/EXAMINATION

A 50‐year‐old healthy male was scheduled for elective left thyroid lobectomy for a large mixed nodule. Preoperatively, the patient showed no clinical signs of airway compromise. Bag mask ventilation was uncomplicated, and direct laryngoscopy with a Macintosh blade size of four was performed without any difficulty resulting in a Cormack and Lehane grade 2 view. The surgery was uneventful.

On post‐operative day 1, the patient developed dysphonia. The upper airway was considered permeable by the surgical team, and when a post‐operative cervical hematoma was suspected, the decision was made to proceed to surgical drainage. Upon arrival in the operating room, the patient received 4 L/min oxygen via a nasal cannula and presented with mild tachypnea and dysphonia.

## 3 METHODS

**FIGURE 1:**
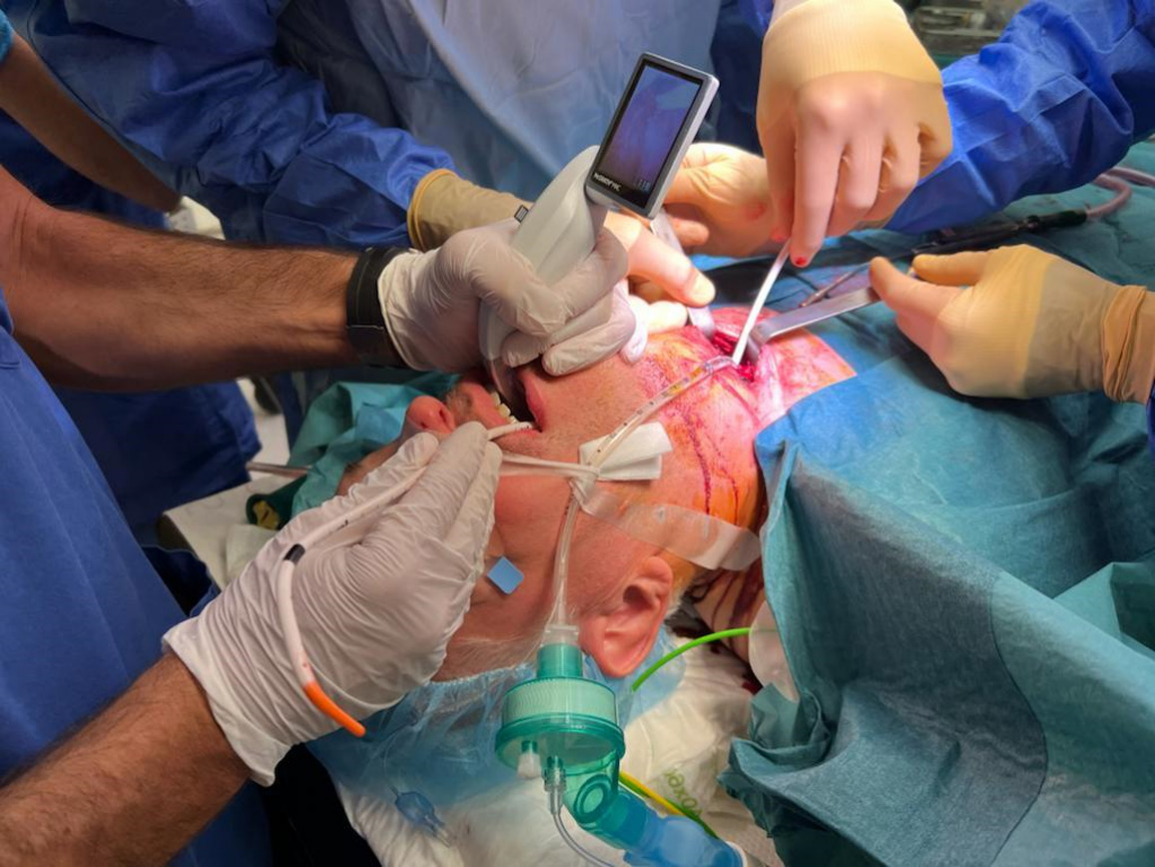
The S‐guide [VBM Medical] was seen exiting the patient's mouth after retrograde insertion through the tracheostoma. The 5.0 orotracheal tube was seen exiting the tracheostoma and was used to ventilate the patient throughout the procedure. Direct visual control of the technique was performed using the McGrath™ MAC video laryngoscope (Medtronic).

**FIGURE 2:**
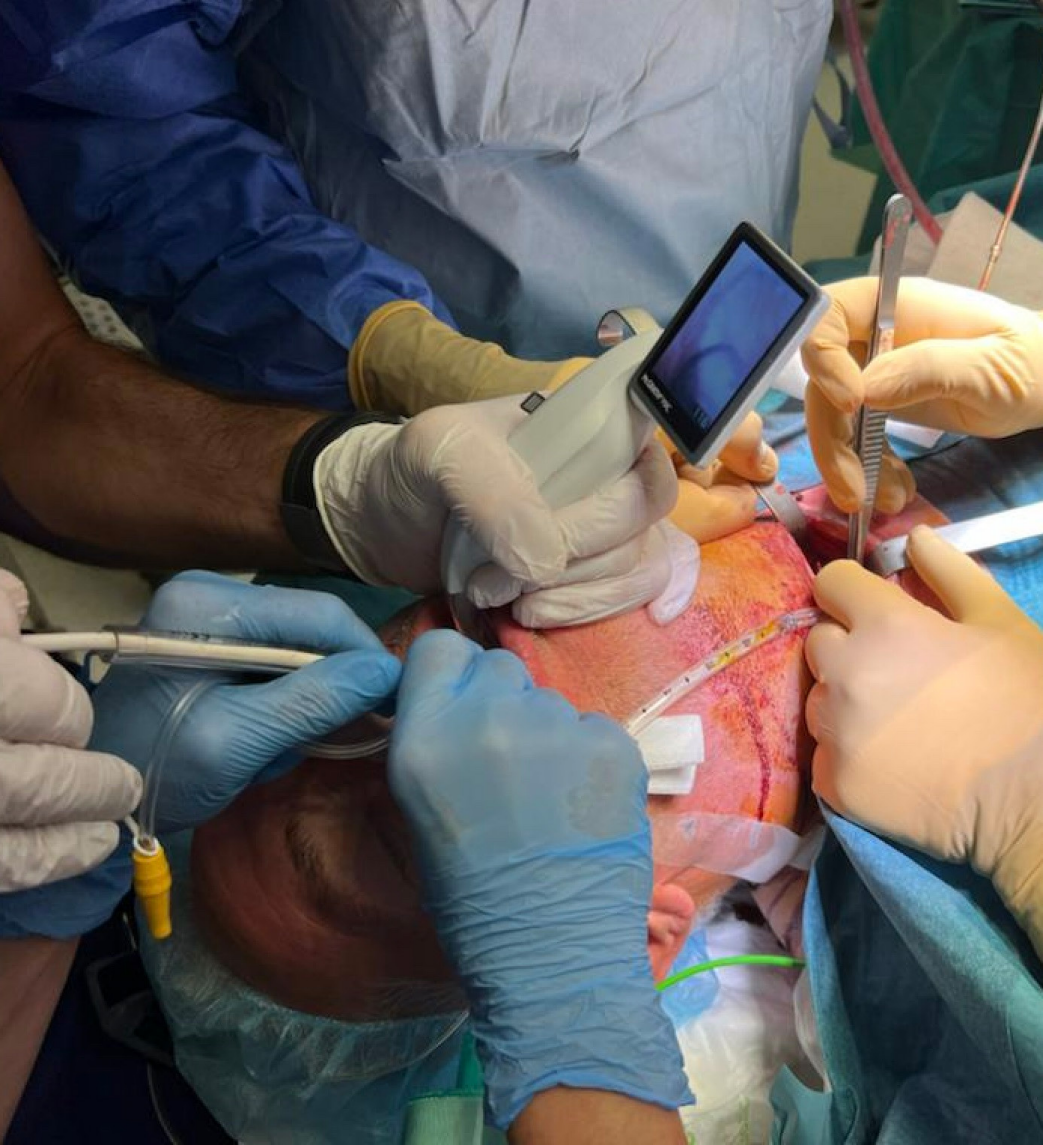
The definitive 6.5 mm orotracheal tube was slid over the S‐guide [VBM Medical] from the mouth until it was visible from the tracheostoma.

Given the high probability of difficult intubation, the strategy is quickly defined by an attending anesthesiologist on site, consisting of several successive steps should the initial plan fail. The patient was placed in a sniffing position, and we decided to proceed with a rapid sequence induction after adequate pre‐oxygenation with 100% inspired oxygen. A McGrath™ MAC video laryngoscope [Medtronic] with a hyperangulated X3‐Blade was chosen for the first attempt. This revealed an edematous airway without the possibility of distinguishing the glottis. Following our local guidelines, an ear, throat, and nose (ENT) surgeon was called, and bag‐mask ventilation was attempted without success. A CICO situation was declared. In light of the airway status, the team decided not to insert a laryngeal mask and immediatly openened the surgical wound just before the ENT surgeon proceeded to an emergent tracheotomy. An endotracheal‐sized 5 mm cuffed tube was inserted through the tracheostoma, and the patient was successfully ventilated. The patient's oxygen saturation remained above 88% during the entire event. The surgical team drained the cervical hematomas. Anticipating post‐operative care, it was agreed to secure the airway using conventional orotracheal intubation in order to improve healing of the cervical tissues and facilitate patient's recovery. After two unsuccessful attempts at indirect glottic visualization with a McGrath video laryngoscope and a flexible endoscope performed by an attending anesthesiologist and a senior ENT surgeon, it was therefore considered that anterograde intubation was technically impossible in this context. In order to achieve conventional orotracheal intubation for the wound healing issues discussed above, a retrograde intubation was attempted. In a situation of swollen and distorted anatomical structures, knowing that retrograde catheterization of the trachea up to the oropharynx would be difficult using the conventional method of a catheter (described below), we decided to use an S‐guide intubation guide. We opted for the S‐guide because of its familiarity to the team (anterogradely) and because it was readily available in the operating theater. The flexible tip of the device was also seen as an advantage as it would avoid further injury to the vocal cords. The flexible tip of the device, which could avoid further injury to the vocal cords, was also seen as an advantage. Despite tissue swelling following regional hemorrhage, the team in charge managed to catheterize the distal trachea up to the mouth under direct visual control of the upper airway using a McGrath video laryngoscope (Figure 1). A size 6.5 mm orotracheal tube was then inserted anterogradely using the S‐guide (Figure 2). When the position of the anterogradely inserted tube was visually confirmed through the tracheostoma, just above the 5 mm tube already in place, the cuff was inflated, and the S‐guide was withdrawn via the mouth. The tracheotomy tube was removed after confirmation of waveform capnography from the anterogradely placed endotracheal tube. The patient's oxygen saturation never dropped during the entire procedure.

## 4 OUTCOME AND FOLLOW‐UP

After 48 h of corticosteroid therapy and 96 h of mechanical ventilation, the patient met all extubating criteria and was therefore extubated without any complications. The patient then described 3–4 weeks of odynophagia, voice fatigue, and difficulty producing high‐pitched sounds. One month later, the ENT examination only described possible hypomobility of the left vocal cord. Clinical control after 8 months showed no sequelae, and the patient had no complaints or dysphonia. Being very athletic, the patient was able to resume his activities and run a full marathon 11 months post‐operatively.

## 5 DISCUSSION

The management of suspected cervical hematoma after thyroid surgery benefits from the guidelines published in 2022.^6^ Except for mild tachypnea, our patient showed no signs of the “DESATS” acronym (*Difficulty swallowing/discomfort; Early warning score; Swelling; Anxiety; Tachypnoea/difficulty breathing and Stridor*) described in the guidelines above, which was set up to help pick up early signs that need urgent senior clinical management. However, we recognized the developing dysphonia as alarming and anticipated the risk of possible difficult or impossible intubation. We prepared a strategy with several plans, including emergency front‐of‐neck access, in case the other steps failed, which was indeed the case. As the staff present was frequently trained in the front‐of‐neck approach and ENT surgeons were readily available in our operating theater, the latter were not involved prior to anesthesia induction. In retrospect, oxygenation may have improved in our management using high‐flow nasal oxygen (HFNO).

Once the airway was secured with tracheotomy and the cervical hematoma was drained, it was reasonable to secure the airway anterogradely. Indeed, after discussion with the surgical team, it was decided that the removal of any foreign device (in this case: the tracheal tube) from the recently operated cervical tissues would help and optimize their healing, as well as facilitating the outcome for the patient. But this proved difficult using indirect laryngoscopy (video laryngoscope and flexible endoscopy). Therefore, we chose retrograde intubation as the best option.

The retrograde intubation technique was first reported by two ENT surgeons in 1960.^7^ It was aimed at ENT cancer surgery by placing an 18G catheter into a tracheostoma and using it as a wire to direct an orotracheal tube anterogradely. Later, in 1963, Waters described an alternative method.^8^ The modified procedure consisted of puncturing the cricothyroid membrane with an 18‐gauge needle, inserting a central venous catheter guidewire until it spontaneously exited the mouth or nose, and then using it as a guide to slide an endotracheal tube until it met resistance against the trachea. The guidewire was then removed, and the tube was inserted deeper into the trachea. Complications associated with retrograde intubation include hemorrhage from the injury to the cricothyroid artery, subcutaneous emphysema, pneumomediastinum, and failure to retrieve the guidewire through the mouth or nose.^9^ In our case, the patient presented with possible left vocal cord hypomobility, had difficulty producing high‐pitched sounds, and complained of voice fatigue 1 month after the events. This could have been a complication of the initial surgery, as well as a complication of airway management during attempts at anterograde or retrograde intubation. A recent international study on airway management training reported that anesthesiologists' trust in this technique is low, prompting the need for further education and training.^10^ Retrograde intubation is an old procedure that is rarely used in the contemporary management of difficult airways, especially because of the widespread use of video laryngoscopes and flexible endoscopes. However, it remains an interesting alternative technique, especially in cases of poor visualization of the glottis anterogradely or in cases of bleeding and pharyngeal secretions.^11^ Retrograde intubation is still recommended as an alternative technique in the 2022 American Society of Anesthesiologists Practice Guidelines for the Management of Difficult Airway.^2^

**FIGURE 3:**
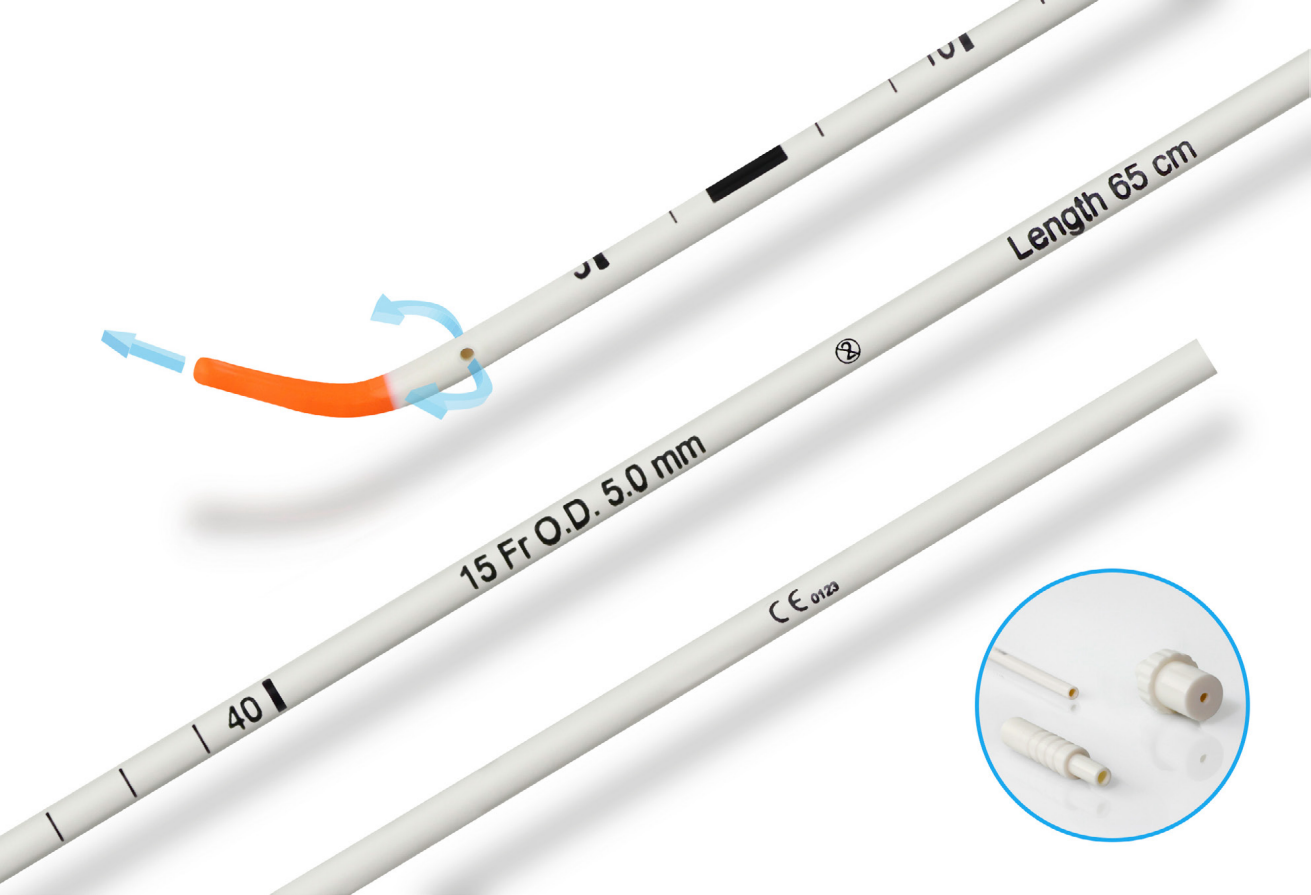
S‐guide intubation guide [VBM Medical]. The distinctive feature of this device is a malleable rigid body combined with a soft atraumatic tip and holes for rescue oxygenation. Courtesy of VBM Medizintechnik GmbH.

**FIGURE 4:**
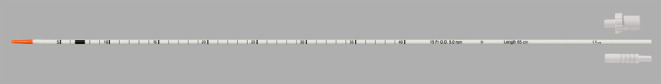
S‐guide intubation guide (VBM Medical) has length marks to easily identify the insertion depth. Courtesy of VBM Medizintechnik GmbH.

In this case, we described retrograde tracheal intubation using an S‐guide intubating guide as an alternative to a central venous catheter guidewire. The novelty we are describing lies in the use of an intubation guide, a well‐known piece of equipment used for tracheal intubation and available in most operating theaters, to perform a procedure (retrograde intubation) that is not frequently performed and sometimes rendered difficult by the use of a conventional central venous catheter guidewire. The S‐guide (Figures 3 and 4) was conceived as an aid for difficult intubation via the anterograde pathway.^12^, ^13^ The distinctive feature of this device is a malleable rigid body combined with a soft atraumatic tip and holes for rescue oxygenation. It also has length marks to easily identify the insertion depth. In our case, the catheter or classical gum elastic bougie seemed too malleable to advance through the severely swollen upper airway. We therefore decided to use the S‐Guide for several reasons specific to an airway that was severely traumatized. The specific configuration of a malleable extremity with a rigid body helping us to get across the engorged and recently bleeding structures, its soft tip to prevent further injury to the vocal cords and tissues, its immediate availability in our operating room, and the impossibility of intubation with a flexible endoscope or a video laryngoscope in an anterograde fashion were the reasons that led us to use it to facilitate retrograde intubation. This unforeseen and innovative use of the S‐Guide as a retrograde intubation guide appears to be a feasible alternative to the conventional technique using a central venous catheter guidewire. Note that the choice of this off‐label use was made by an experienced team that was fully aware of the limitations of the chosen strategy. Hence, the success of the technique described in this case report may encourage further research.

Several learning points emerge from this case report. First, that front of neck access remains the standard and expected emergency procedure in the event of a CICO situation. Second, that the technique of retrograde intubation should be known, even if its use remains anecdotal today, as it can be used to tunnel and guide the airway in specific situations. Third, in a situation where the airway is traumatized and swollen, an S‐Guide intubating guide may be a useful alternative to the classical central venous catheter guidewire.

In conclusion, in a CICO situation, once an emergency front‐of‐neck access has allowed for patient oxygenation, retrograde intubation can be an effective way of securing the airway when anterograde endotracheal intubation techniques have been unsuccessful. The use of a S‐Guide intubating guide may be a useful alternative to achieve this procedure.

## AUTHOR CONTRIBUTIONS

**Fiona Ollier:** Conceptualization; validation; writing – original draft; writing – review and editing. **Alain‐Stéphane Eichenberger:** Validation; writing – review and editing. **Georges L. Savoldelli:** Supervision; validation; writing – review and editing. **Julien Maillard:** Conceptualization; funding acquisition; project administration; resources; supervision; validation; writing – original draft; writing – review and editing.

## FUNDING INFORMATION

None.

## CONFLICT OF INTEREST STATEMENT

The authors declare that they have no conflicts of interest regarding the publication of this article.

## CONSENT

Written informed consent was obtained from the patient to publish this report in accordance with the journal's patient consent policy.

## Data Availability

The data used to support the findings of this study are included within the article.
